# Perventricular device closure of a large residual perimembranous interventricular septal defect after previous surgical correction

**DOI:** 10.1186/1749-8090-9-12

**Published:** 2014-01-10

**Authors:** Edvin Prifti, Arben Baboci, Edvin Dado, Efrosina Kajo

**Affiliations:** 1 Division of Cardiac Surgery, University Hospital Center of Tirana, Tirana, Albania; 2 Division of Cardiac Surgery, University Hospital Center of Tirana, Rr. Dibres, 370, Tirana, Albania

**Keywords:** Perventricular device closure, Perimembranous ventricular septal defect

## Abstract

A 21 years albanian patient was referred with important residual left to right shunt. He was undergone 7 years before conventional surgical correction of a perimembranous ventricular septal defect (VSD). The patient underwent sternotomy and perventricular device closure of the residual employing a 16 mm multifenestrated atrial septal defect occlude, which was positioned through the anterior wall of the right ventricle. across the defect. The previous autologous pericardial patch was compressed into the double umbrella device. We may conclude that perventricular device closure can employed successfully in patients with residual perimembranous VSD after previous surgical repair as an alternative to the conventional surgery with excellent hemodynamic and postoperative outcome. Such a technique should be part of the surgical armamentarum.

## Background

Perventricular device closure of ventricular septal defect (VSD), as an alternative technique has been initially reported in 1999 in laboratory animals [1] and later in patients with perimembranous VSD [2]. Such a technique seems to be a safe alternative to conventional surgical correction making possible the closure of perimembranous VSD without cardiopulmonary bypass. However the employment of PDC in residual perimembranous VSD after previous surgical correction is very limited with only one case in the literature [3]. Here we report a patient undergoing PDC of a residual perimembranous VSD after previous surgical repair.

## Case presentation

A 21 years albanian patient was referred to our division. The patient presented dyspnea, fatigue, palpitations. Seven years ago he was undergone surgical correction of a perimembranous VSD employing cardiopulmonary bypass. During the first operation a perimembranous VSD of 15-18 mm and pulmonary stenosis were found. The perimembranous VSD was closed using an autologous patch. The transthoracic echocardiogram showed a significant left-to-right shunt, half systemic right ventricular pressure and dysfunction of the left ventricle. The patch was “floating” with only anterior part of patch was attached to the interventricular septum. A partially detached patch by previous surgical closure of the VSD, with large extension to the inlet portion of the septum, was located.

**Figure 1:**
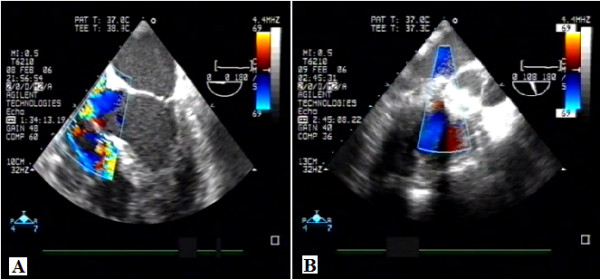
**Intraoperative transesophageal echocardiography pre and after device implantation. A**. Intraoperative transesophageal echocardiography demonstrating an important left to right shunt and **B**. Intraoperative transesophageal echocardiography demonstrating non residual left to right shunt after the device implantation.

**Figure 2:**
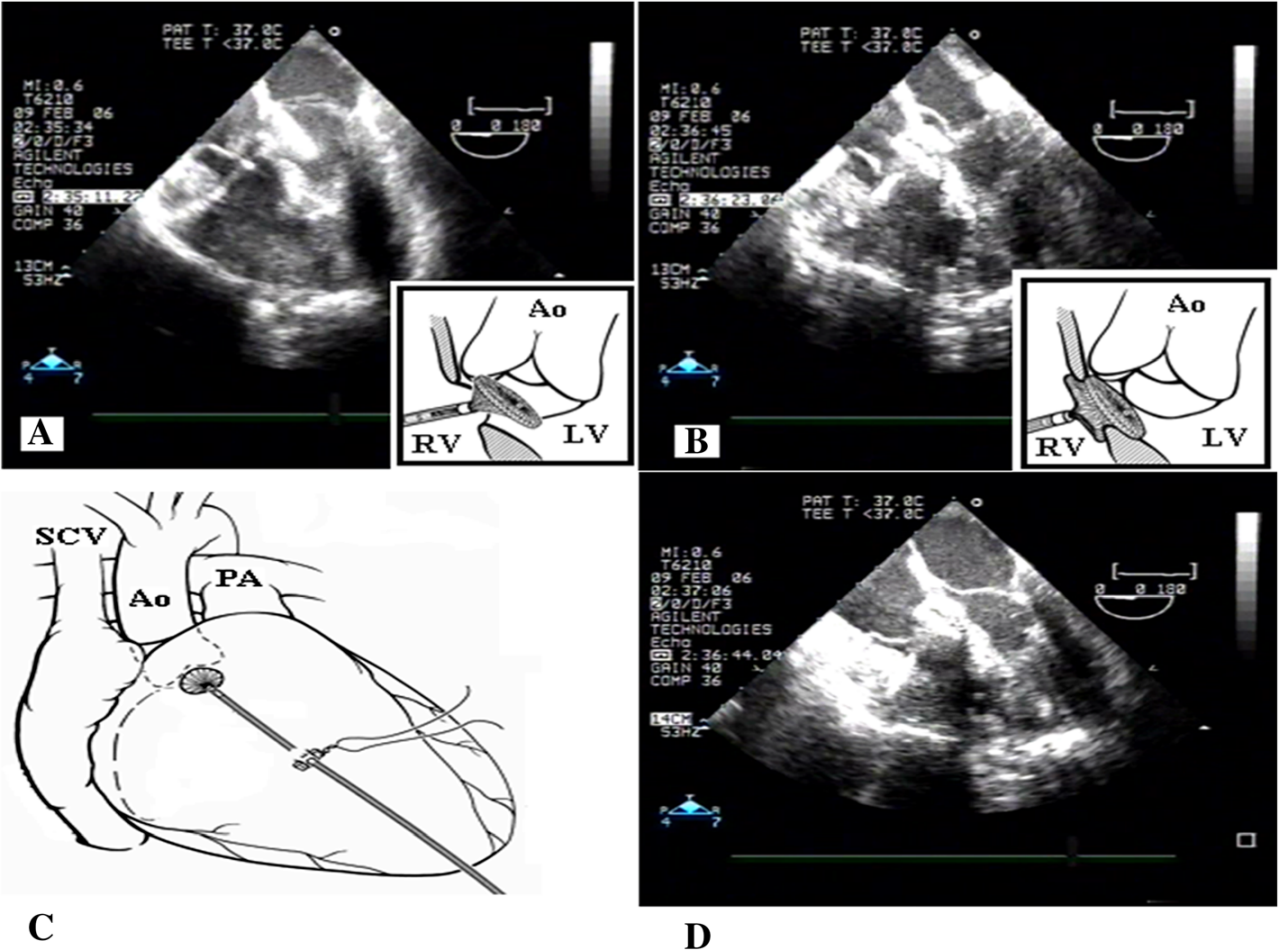
**Technical aspects of the preventricular device implantation technique. A**. Insertion of the device, opening of the first hemiumbrella on the left side of the ventricular septal defect. **B**. Opening of the right hemiumbrella on the right side of the ventricular septal defect. **C**. Insertion of the delivery system through the anterior wall the right ventricle. **D**. The device already positioned and removal of the delivery system. Legend: Ao-Aorte, PA-Pulmonary artery, SCV-Superior Cava Vein, LV-Left ventricle, RV-Right ventricle

The patient had a clear indication for residual VSD closure. During re-sternotomy, heavy adherences were found. The decision was made to proceed with PDC. Transesophageal echocardiography was used in tandem to guide direct needle puncture of the right ventricular free wall, taking care to avoid right coronary marginal branches (Figure 1A). A puncture site perpendicular to the plane of the VSD was selected (Figure 2C). A pledgetted 4–0 Prolene purse-string suture was placed around the chosen puncture site. A flexible guide wire was introduced into the right ventricle through a 20-gauge needle and was maneuvered into the left ventricle through the defect; the needle was then removed. Using the modified Seldinger technique, a 9 French sheath was inserted into the right ventricular and directed across the VSD using a J tipped short wire. A purse string suture was placed around the sheath. Size estimates of the defect diameter by 2D-echo with color flow Doppler measured 14 mm at the major extension and appeared to be predominantly in the postero-inferior portion of the perimembranous VSD. However, the defect was within a few millimeters of the anterior mitral leaflet in diastole and aortic valve. The delivery system was pushed very high and posteriorly passing under the tricuspid septal leaflet. A 16-mm multifenestrating atrial septal defect occluder (Amplatzer) was then positioned across the defect. When the delivery system was under the aortic valve, we opened the first hemiumbrella (Figure 2A) and we found the right place for opening the second disk controlling both tricuspid and aortic valve (Figure 2B). The device was easily delivered and transesophageal echocardiography demonstrated a good position of the double umbrella without damaging the tricuspid and aortic valve. The patch was compressed into the double umbrella device (Figure 2D). No residual shunt were detected immediately after the procedure (Figure 1B). At three years follow-up the patient is doing well without any problem confirming the good position of the device.

## Conclusions

VSD are classified according to their location within the septum, however the most common type is the perimembranous type. Indications for perimembranous VSD closure are symptoms of heart failure, signs of left heart chambers volume overload, or a history of endocarditis. In patients with a volume overload of the left heart, closure is necessary to prevent pulmonary arterial hypertension, ventricular dysfunction, and arrhythmias.

The surgical approach is considered to be the gold standard, but it is associated with morbidity and mortality and the use of cardiopulmonary bypass. Percutaneous techniques have been developed, however possible complications of such a procedure might be the embolization or displacement of the device, arrhythmias, the hemolysis, the valvular insufficiency that can interest the aortic valve, tricuspid or mitral, pericardial effusion or the cardiac tamponade.

The most greater limit of the transcatheter closing of the VSD appeared in the approach to the perimembranous defects, for the risk of damage of the aortic valve: new techniques and new devices have allowed to also overcome this obstacle. Such risks increases significantly in residual VSD after previous surgical closure.

PDC is another alternative for perimembranous VSD closure, and initial reports have demonstrated excellent outcome of such a technique [2,4,5]. From the morphological aspect, a part of the margin of a perimembranous VSD is the area of fibrous continuity adjacent to the central fibrous body that bears the atrioventricular conduction bundle. These defects are in proximity to the aortic, tricuspid, or mitral valves. The previous studies have demonstrated a very low heart block rate [4,5] differently to the percutaneous approach. Possible explanations may be device related, or delivery related. Whereas percutaneous techniques require a tortuous catheter path through the heart and ultimately through the VSD, the PDC approach allows for a device placement just from the “opposite” right ventricular free wall. Neither guidewires nor sheaths need to pass through in an angle, push and pull on perimembranous VSD margins.

The chosen device needs to be oversized as in our case by 2 millimeters. Since there was an extremely short route to the “area of interest”, a small step-up in size of the device allowed for a snug closure of the defect. Since the device had to close itself along the imaginary line (the line of the detached patch) passing from the posterior margin of perimembranous VSD, below the septal leaflet of the tricuspid valve and the subaortic upper point, we had to choose a device who could have a relatively small axis and a large biplane dimension as a multifenestrating atrial septal defects occluder (Amplatzer®).

We may conclude that PDC can be employed successfully in patients with residual perimembranous VSD after previous surgical repair as an alternative to the conventional surgery with excellent hemodynamic and postoperative outcome. Such a technique should be part of the surgical armamentarum.

## Consent statement

Written informed consent was obtained from the patient for publication of this case report and any accompanying images. A copy of the written consent is available for review by the Editor-in-Chief of this journal.

## Abbreviations

VSD: Ventricular septal defect; PDC: Perventricular device closure.

## Competing interests

The authors declare that they have no competing interests.

## Authors’ contributions

All authors read and approved the final manuscript. Dr. EP carried out the case as the first surgeon, and drafted the manuscript. Dr. AB, was the assistant surgeon during the procedure. Dr. ED, is the invasive hemodynamist supported us during the hybrid procedure and Dr. EK, performed the transesophageal echocardiography during and after the procedure and the follow-up of the patients, providing the echo images.

## References

[B1] Amin Z Gu X Berry JM Titus JL Gidding SS Rocchini AP Perventricular [correction of Periventricular] closure of ventricular septal defects without cardiopulmonary bypass Ann Thorac Surg 1999 9 1 149 153 discussion 153–4 10.1016/S0003-4975(99)00519-6 10421131

[B2] Zeng XJ Sun SQ Chen XF Ma XJ Luo YH Lim YP Tao L Device closure of perimembranous ventricular septal defects with a minimally invasive technique in 12 patients Ann Thorac Surg 2008 9 1 192 194 10.1016/j.athoracsur.2007.07.018 18154808

[B3] Aboulhosn J Levi D Sopher M Johnson A Child JS Laks H Perventricular closure of a large ventricular septal defect in congenitally corrected transposition of the great arteries Congenit Heart Dis 2010 9 1 60 65 10.1111/j.1747-0803.2009.00339.x 20136860

[B4] Pan S Xing Q Cao Q Wang P Duan S Wu Q Hou K Perventricular device closure of doubly committed subarterial ventral septal defect through left anterior minithoracotomy on beating hearts Ann Thorac Surg 2012 9 6 2070 2075 10.1016/j.athoracsur.2012.05.070 22921238

[B5] Zhu D Gan C Li X An Q Luo S Tang H Feng Y Lin K Perventricular device closure of perimembranous ventricular septal defect in pediatric patients: technical and morphological considerations Thorac Cardiovasc Surg 2013 9 4 300 306 10.1055/s-0033-1334997 23564538

